# Application of Gold-Nanoparticle Colorimetric Sensing to Rapid Food Safety Screening

**DOI:** 10.3390/s18124166

**Published:** 2018-11-27

**Authors:** Guangyang Liu, Meng Lu, Xiaodong Huang, Tengfei Li, Donghui Xu

**Affiliations:** 1 Institute of Vegetables and Flowers, Chinese Academy of Agricultural Sciences, Key Laboratory of Vegetables Quality and Safety Control, Ministry of Agriculture and Rural Affairs of China, Beijing 100081, China; liuguangyang@caas.cn (G.L.); 18230106173@163.com (M.L.); huangxiaodong@caas.cn (X.H.); 2 College of Life Sciences and Engineering, Hebei University of Engineering, Handan 056021, China

**Keywords:** gold nanoparticles, colorimetric sensor, synthesis, functional modification, optical property, food safety

## Abstract

Due to their unique optical properties, narrow size distributions, and good biological affinity, gold nanoparticles have been widely applied in sensing analysis, catalytic, environmental monitoring, and disease therapy. The color of a gold nanoparticle solution and its maximum characteristic absorption wavelength will change with the particle size and inter-particle spacing. These properties are often used in the detection of hazardous chemicals, such as pesticide residues, heavy metals, banned additives, and biotoxins, in food. Because the gold nanoparticles-colorimetric sensing strategy is simple, quick, and sensitive, this method has extensive applications in real-time on-site monitoring and rapid testing of food quality and safety. Herein, we review the preparation methods, functional modification, photochemical properties, and applications of gold nanoparticle sensors in rapid testing. In addition, we elaborate on the colorimetric sensing mechanisms. Finally, we discuss the advantages and disadvantages of colorimetric sensors based on gold nanoparticles, and directions for future development.

## 1. Introduction

Recently, food quality and safety issues have attracted much attention in China and the rest of the world. Specific recognition and effective detection of contaminants in food is very important for controlling and monitoring food quality and safety incidents during food consumption. The main detection technologies for food quality and safety are instrumental analysis and rapid tests. The instrumental methods, such as gas chromatography [1], liquid chromatography [2], mass spectrometry (MS) [3], capillary electrophoresis [4], supercritical fluid chromatography [5], gas chromatography-MS [6], and liquid chromatography-MS [7], are undoubtedly powerful. All of these methods possess high sensitivity, good accuracy, and high stability, but they are often inconvenient, expensive, time-consuming, and require large, expensive analytical instruments and trained operators [8]. For effective target recognition, rapid test technologies require high selectivity for the target and high sensitivity. Rapid tests have been emerged as one of the most promising techniques in food quality and safety analysis because they use antibodies, enzymes, and aptamers for the molecular recognition. Established methods include the enzymatic inhibition method [9], ELISA biosensing technique [10], surface plasmon resonance immunosensors [11], and electrochemical immune sensors [12]. However, all these methods have problems, such as difficulties with antibody preparation, complicated enzyme purification methods, severe matrix interference, and poor stability and repeatability.

Pesticide residues, heavy metals, biotoxins, and banned additives in food are toxic to humans and there is an urge need to monitor them in food safety. In recent decades, with the rapid development of materials science and nanotechnology, a lot of new nanocomposites with different molecular recognition and signal transduction properties are constantly emerging. These materials have been widely applicated in specifically, rapidly and accurately detect chemical contaminants in food.

**Figure 1:**
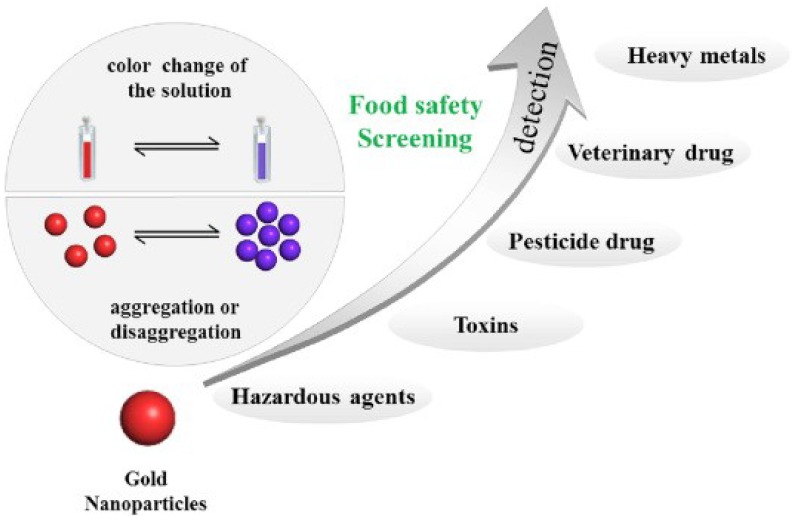
Application of gold nanoparticle colorimetric sensors in food safety screening. Reproduced with permission from reference [30].

Gold nanoparticles, usually with a particle size distribution between 1 nm and 100 nm, also known as colloidal gold, are very stable nanomaterials that have been widely investigated and applied [13,14,15]. Because of their unique physicochemical properties, gold nanoparticles have been used in chemical energy, electronic devices, environmental monitoring, biomedicine, and food safety screening [16,17,18]. Their beneficial properties for electronic, catalytic, chemical, and optical properties have made them popular in research on biochemical sensing technologies, immune analysis, electrochemical analysis, and biomedicines [19,20,21,22,23,24]. Surface plasmon resonance properties of gold nanoparticles cause their maximum characteristic absorption peak wavelength to shift in the UV-visible region with changes in the particle size, morphology, and interparticle distance, and this is accompanied by a color change [25,26,27]. Gold nanoparticles could be used to establish optical sensing technologies using aggregation (or deaggregation) of the nanoparticles induced by formation of covalent or non-covalent bonds with the target substance. The solution of gold nanoparticles will be changed from wine red to blue caused by the agglomeration of gold nanoparticles, corresponding with surface plasmon band shifts from 523 nm towards 610~670 nm [28,29,30]. In addition, functional gold nanoparticles based on the target analyte-triggered preparation can be used to sense several hazards in food [31,32,33]. Colorimetric sensors based on gold nanoparticles are simple, rapid, and highly sensitive, and have been widely applied in real-time on-site monitoring and rapid testing of food quality and safety. The application of gold nanoparticle colorimetric sensors in food safety screening was illustrated in Figure 1.

## 2. Synthesis of Gold Nanoparticles

The unique physicochemical properties of gold nanoparticles have attracted increasing attention from scientists. The preparation methods of gold nanoparticles are mainly divided into physical and chemical methods [34,35]. Physical methods include vacuum condensation, electrical dispersion, ultrasonication, and laser ablation [36]. Physical methods allow for control of the gold nanoparticle shape, but large equipment is often needed. Moreover, the methods are complicated, and the nanoparticle size distribution is not uniform. Chemical methods include citric acid reduction, the sulfhydryl ligand method, seed crystal growth, and electrochemical and photochemical methods [37]. Among the chemical methods, the first three listed above are commonly used.

Citric acid reduction, also known as the Turkevich–Frens method, is the most commonly used method, and is a simple water-phase oxidation-reduction method that was first described by Turkevich [38]. First, a chloroauric acid solution of a certain concentration is boiled, and a certain amount of sodium citrate solution is added. The Au(III) is reduced and aggregates to forms gold cores. Through electrostatic interactions, ions (H^+^, AuCl_4_^−^) adsorb on the surfaces of the cores and form a stable colloidal gold solution [39,40]. As well as acting as a reductant, sodium citrate can be used as a ligand to modify the surfaces of the gold nanoparticles to prevent aggregation and precipitation [41]. The method is simple, and the prepared gold nanoparticles are well dispersed with a uniform particle size distribution. The particle size is affected by the ratio of chloroauric acid to sodium citrate, and can be controlled within 3–100 nm by changing the ratio [42].

In the sulfhydryl ligand method, HAuC1_4_ and sulfhydryl ligands are added into a double- or single-phase solvent. Then, a strong reducing agent (sodium borohydride) is added to reduce the Au ions to gold nanoparticles. The sulfhydryl ligands can bind to the gold nanoparticle surface through Au–S bonds [43]. Gold nanoparticles prepared by this method often have narrow size distributions that are generally within 0.8–8 nm. According to the polarity of solvent, this method is classified as single- or double-phase. In the single-phase method [44], a water-soluble sulfhydryl ligand such as thioglycolic acid, cysteine, or glutathione, is used as a ligand to prepare gold nanoparticles that have good dispersity in water. In the double-phase method [45], alkyl sulfhydryl is often used as a ligand, and the prepared gold nanoparticles possess good oil solubility and are stable in organic solvents. The Brust–Schiffrin method [46] is a classic sulfhydryl ligand method that uses tetra-*n*-octylammonium bromide as an agent to transfer chloroauric acid from the water phase into the organic phase. Then NaBH_4_ and thiol are used as the reducing agent and ligand, respectively, to prepare gold nanoparticles. tetra-*n*-Octylammonium bromide can be used as a phase transfer agent and stabilizer to protect the gold nanoparticles [47].

In the seed growth method, small gold nanoparticles prepared by water-phase oxidation-reduction are used as seeds, a chloroauric acid solution is used as a growth solution, and HAuCl_4_ is reduced on the surfaces of the gold nanoparticles, which produces large gold nanoparticles [48]. Particles with different sizes and morphologies, such as spherical, rod-like, and triangular, can be prepared by controlling the ratio of crystal to growth solution [49]. El-Sayed et al. [50] used gold nanoparticles modified by cetyltrimethylammonium bromide as seed crystals and a monovalent gold nanoparticle solution as the growth solution to prepare gold nanorods with controlled axial ratios. 

## 3. Functional Modification of Gold Nanoparticles

**Figure 2:**
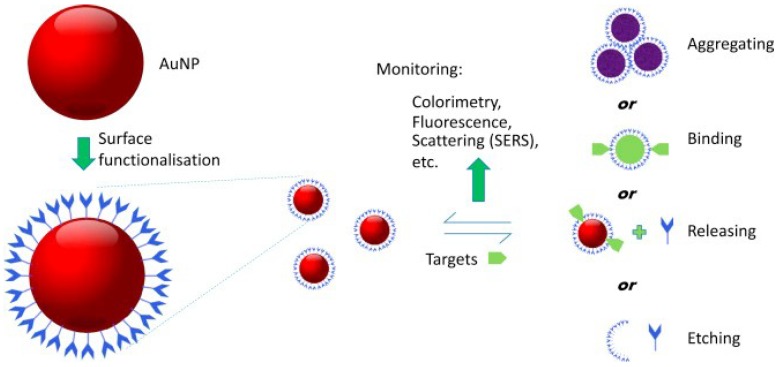
Gold nanoparticle-based sensing in analytical science. Reproduced with permission from reference [51].

Because of their surface properties, the gold nanoparticles obtained directly from these preparation methods do not meet the requirements for chemical or biological sensing. Therefore, their surfaces need to be modified with functional groups with high selectivities, surface activities, and binding capabilities to improve the nanoparticle dispersity and compatibility [51], the strategies of surface modification techniques were shown in Figure 2.

Molecular modification of the surfaces of gold nanoparticles increases their stability and improves their physicochemical properties and functionality, which means they can be used in more biological chemical reactions to expand their application [52]. Current surface modification methods use covalent bonding [53,54], electrostatic interactions [55], or attachment of a small molecule or compound for specific recognition of the target [56].

**Figure 3:**
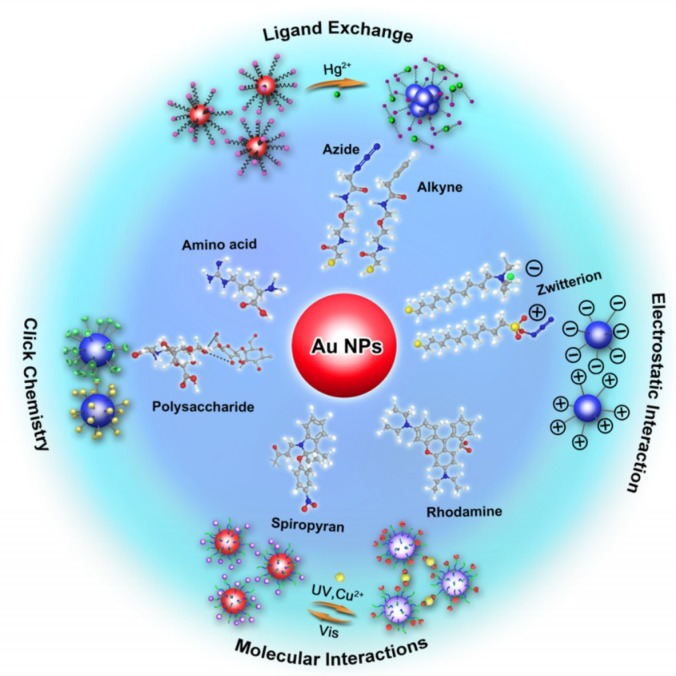
Strategies for functionalization of gold nanoparticles (AuNPs) with small molecules. Reproduced with permission from reference [52].

In covalent modification, the surfaces of the gold nanoparticles are modified with functional biological or organic molecules containing sulfhydryl groups via Au–S bonds. By optimizing the ratio of ligand to nanoparticle and type of sulfhydryl ligand, gold nanoparticles with multiple functions can be prepared. For example, when single stranded DNA and antibody were introduced onto the surface of AuNPs, endows themselves high specific recognition ability for antigens and DNA complementary strand simultaneously [11]. Therefore, a novel AuNPs with multiple recognition ability will be synthesized through covalent modification. The interactions between gold nanoparticles and sulfhydryl ligands are strong, and this modification improves the stabilities of gold nanoparticles under high salt, acid, or base conditions, and at high temperatures. However, multiple procedures are often needed to obtain sulfhydryl ligands with specific functions, which leads to complicated preparation methods. The commonly used sulfhydryl ligands include thiol [57], thioglycolic acid [58], cysteamine [59], cysteine [60], glutathione [61], protein [62], and acetic acid [63]. Strategies for functionalization of gold nanoparticles (AuNPs) with small molecules were summarized in Figure 3.

Electrostatic modification involves modification of the nanoparticle surface with ligands via electrostatic interactions [64]. Compared with covalent bonds, electrostatic interactions are weak and the modified nanoparticles have poor stabilities. Under high salt or strong acid/base conditions, the ligands are easily removed, resulting in aggregation of the gold nanoparticles [65].

Moreover, the functionalization of gold nanoparticles with antibody gives the specific recognition and detection of the target antigen molecules in matrix [66,67]. For instance, streptavidin-gold nanoparticles can specifically bind biotinylated antibody through streptavidin-biotin bonding interaction [68]. And glycosylated gold nanoparticles applied for recognizing immunoglobulin with high sensitivity and selectivity [69].

## 4. Optical Properties of Gold Nanoparticles

Because of their small sizes, gold nanoparticles have many unique physicochemical properties, such as good optical characteristics, high electron densities, and good biocompatibility and catalytic performance [70]. Their surface plasmon resonance [71] and fluorescence quenching [72,73] are the most applied optical properties.

**Figure 4:**
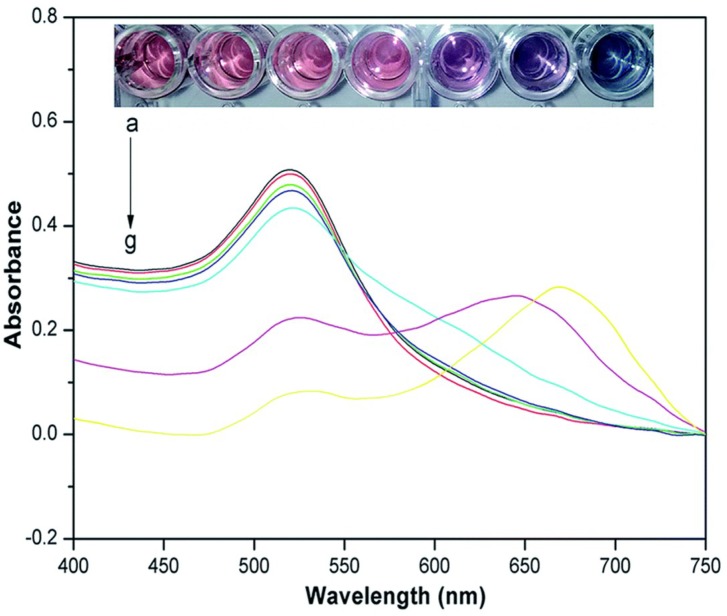
Changes in the colors and UV-visible spectra of a cysteamine–gold nanoparticle solution with different concentrations of atrazine. Reproduced with permission from reference [55].

Surface plasmon resonance occurs when the vibrational frequency of free electrons interacts with the frequency of incident light to generate resonance coupling [51]. This causes a strong characteristic absorption for gold nanoparticles in the UV-visible region. The maximum absorption wavelength shifts with changes in the sizes, shapes, and interparticle distances of the gold nanoparticles [74]. For example, 13-nm gold nanoparticles have a maximum absorption wavelength at 523 nm and are wine red. An increase in the particle size or decrease in the interparticle distance causes a redshift of the maximum adsorption wavelength and a color change (faint yellow, wine red, purple red, blue) [55,75,76]. Liu et al. [55] developed a simple and sensitive colorimetric sensor to detect atrazine by using cysteamine–gold nanoparticles, changes in the colors and UV-visible spectra of a cysteamine–gold nanoparticle solution with different concentrations of atrazine were shown in Figure 4.

The absorption band of gold nanoparticles in the UV-visible spectrum is relatively wide, and the extinction coefficient increases with increases in the gold nanoparticle size [77]. Because the gold nanoparticle extinction coefficient is much higher than that of traditional chromophores, it can be used as a strong acceptor to quench the fluorescence of the donor [78,79]. This characteristic is often applied in fluorescence resonance energy transfer or inner-filter fluorescence sensing to detect proteins [80,81], nucleic acids [82], pesticide residues [83,84], heavy metal ions [85,86], and environmental pollutants [87,88]. 

## 5. Principle of Gold Nanoparticles-Based Colorimetric Sensors and Application in Food Quality and Safety Testing

Colorimetric analysis of gold nanoparticles is based on their optical properties and the large color changes caused by gold nanoparticle aggregation and changes in their morphologies or interparticle distances [89,90]. Aggregation of gold nanoparticles with appropriate sizes leads to surface plasmon resonance coupling, and causes color changes in the visible region. For example, aggregation of gold nanoparticles (*d* > 3.5 nm) induces interparticle surface plasmon coupling in a gold nanoparticle gel, and it turns from red to blue [91].

Colorimetric sensors contain two critical elements that determine their sensitivity, selectivity, response time, and signal-to-noise ratio [51,92]. One of these element is the recognition unit, which has a selective response, reaction, or interaction with the target compound (e.g., organic small molecule, biomacromolecule) [93]. The other element is the conduction unit, which is a nanomaterial with good optical properties. The conduction unit transforms the detection response into a color change in the visible light region (390–750 nm), and determines the detection sensitivity [94]. According to the mechanism by which the optical properties of the gold nanoparticles are changed, colorimetric analyses can be divided into cross-linking and deprotection methods.

### 5.1. Cross-Linking Methods and Application

In cross-linking methods, the target compound and ligand on the gold nanoparticle surface form a complex, which decreases the interparticle distance and causes aggregation [95]. This method requires nanoparticle modification with specific ligands. Generally, one terminal of the ligand should have a sulfhydryl group and the other should have a functional group that can bind with the target. The sulfhydryl group of the ligand can bind with the surface of a gold nanoparticle through a strong Au–S bond [96]. In the presence of the target compound, the ligand also binds with the target compound and reduces the gold nanoparticle interparticle distance and causes aggregation [97]. The role of the ligand is to draw the nanoparticles closer together. The main interactions resulting in cross-linking aggregation in gold nanoparticle-based colorimetric methods are chelation, chemical bonding, base pairing, electrostatic interactions, and hydrogen bonding [98]. 

In our laboratory, we modified gold nanoparticles with melamine, which acted as the recognition unit to aggregate the gold nanoparticles by forming Au–N bonds [99]. In the presence of atrazine, the interparticle distance decreased because of hydrogen bond formation between melamine and atrazine and the gold nanoparticles in the solution aggregated, leading to surface plasmon resonance and changes in the absorption spectrum and color of the gel. The limit of detection was 165 nM and the detection time was 15 min. Giannoulis et al. [100] used C18 to extract dithiocarbamate, and this removed most of the matrix and other interferences. The sulfhydryl group of dithiocarbamate cross-linked with the gold nanoparticles by formation of Au–S bonds, causing aggregation and a color change. This was used to establish a sensing technique with a linear range of 4.2–42 nM and a limit of detection of 1.05 nM. Gold nanoparticles specifically recognize triadimenol by electrostatic interactions, causing a red shift in the surface plasmon resonance peak, cross-linking aggregation, and a color change. The linear range is 0.338–33.8 μM and the detection time is 10 min [101].

Nan et al. [102] modified gold nanoparticles with Triton X-100, which formed multiple cross-links with melamine, leading to aggregation and a color change. The limit of detection for melamine in milk was 5.1 nM. Turibius et al. [103] prepared gold nanoparticles modified with glutamic acid via electrostatic interactions, and found they had supernormal stability and remained stable at room temperature for 6 months. Hydrogen bonding between glutamic acid and clenbuterol or ractopamine could induce cross-linking aggregation and color changes of gold nanoparticles. The limits of detection for clenbuterol and ractopamine in human urine were 0.23 nM and 0.43 nM, respectively. Scientists have also used gold nanoparticles modified with melamine to specifically recognize ractopamine and salbutamol [104]. The absorbance ratio at 670 nm/520 nm gave a detection sensitivity of 100 nM with a wide linear range. The linear ranges for detection of ractopamine and salbutamol were 0.1–500 nM and 0.1–10 μM, respectively.

Anwar et al. [75] prepared gold nanoparticles modified with thioacetic acid using the Au–S bond. Metal complexation between acetic acid and palladium ions could cause aggregation of the gold nanoparticles and a color change from red to blue, which could be used to detect palladium ions in water and human plasma with a limit of detection of 4.23 μM. Ratnarathorn et al. [105] used gold nanoparticles modified with maleic acid to specifically bind lead ions, and established a colorimetric sensing method with a linear range of 0.0–30.8 nM and limit of detection of 1.54 nM. Wang et al. [106] used melamine that could specifically bind with cadmium and mercury ions to modify gold nanoparticles. When cadmium ions or mercury ions were added, single heavy metal ions interacted simultaneously with multiple cyanuric acid molecules on the gold nanoparticles. This led to cross-linking of the gold nanoparticles and a change in the color of solution from red to blue. Cadmium and mercury ions were detected with sensitivities of 3.5 nM and 2.8 nM, respectively. Chen et al. [107] used gold nanoparticles modified with DMSA to specifically recognize Cr^3+^/Cr^4+^. They combined the nanoparticles with a smart phone to build a rapid test device for detecting Cr^3+^ in soil with a limit of detection of 10 nM.

### 5.2. De-Protection Method and Application

In the deprotection method, free specific aptamers (DNA/RNA fragments, nucleic acid aptamers, or non-sulfhydryl small organic molecules) are mixed with gold nanoparticles and bind to their surfaces through weak interactions. With a high salt concentration, the nanoparticles remain dispersed and are red. Strong binding between the target compound and the aptamer makes the ligand detach from the surface of gold nanoparticles, which causes aggregation and a color change to blue [108]. 

**Figure 5:**
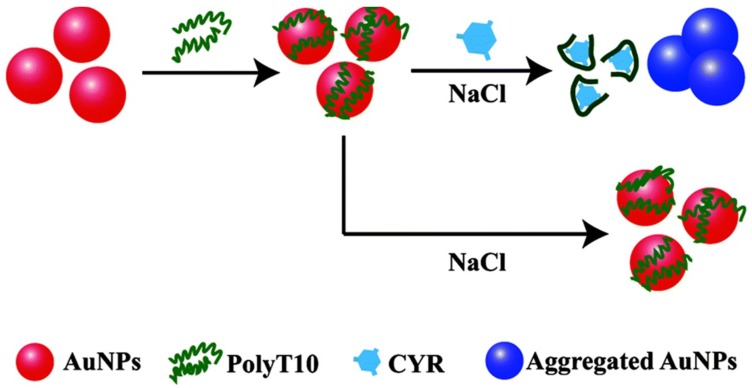
Label-free gold nanoparticle (AuNP) colorimetric sensor for optical detection of cyromazine. Reproduced with permission from reference [110].

Zhang et al. [109] used gold nanoparticles modified with an ionic liquid to detect imidacloprid, which underwent ligand exchange with the ionic liquid. In the presence of imidacloprid, electrostatic interactions occurred, and the gold nanoparticles aggregated and changed color. The limit of detection was 0.5 μM. Liu et al. [110] prepared polythymine-gold nanoparticles, and used them to detect cyromazine in cucumber based on gold nanoparticle aggregation induced by a high salt concentration. The polythymine on the gold nanoparticle surface specifically bound with cyromazine through multiple hydrogen bonds, and was then removed, causing aggregation and a color change under high salt concentrations and the sensing strategy was presented in Figure 5. The linear range was 6.0–3.0 μM and recovery rate was 83.7–104.8%. Yang et al. [111] prepared gold nanoparticles modified by the ochratoxin A aptamer, and used the recognition between the toxin and aptamer to detach the aptamer from the gold nanoparticle surface. Furthermore, the gold nanoparticles aggregated and a color change occurred under high salt concentrations, and the limit of detection was 20 nM. Single-stranded DNA can protect gold nanoparticles from aggregation in a solution with a high salt concentration. Liang et al. [112] and Wu et al. [113] proposed two colorimetric assays using an oligonucleotide with a GT sequence or an aptamer to detect arsenic with sensitivities of 10 nM and 25 nM, respectively. To date, aptamers of various antibiotics have been screened and gold nanoparticle colorimetric rapid tests for sulfadimethoxine [114], penicillin [115], oxytetracycline [116], and streptomycin [117,118] have been established.

### 5.3. Anti-Aggregation Method and Application

**Figure 6:**
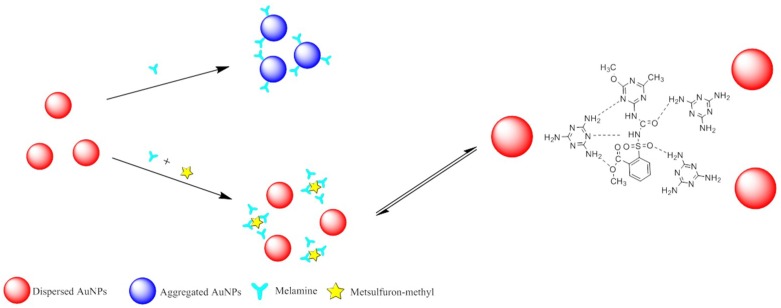
Sensing principle for metsulfuron-methyl analysis based on anti-aggregation of gold nanoparticles. Reproduced with permission from reference [120].

In the anti-aggregation system, the model molecules (or linkers) induce aggregation by cross-linking gold nanoparticles via the formation of linkages. However, the physicochemical forces (e.g., complexation, electrostatic interactions, and hydrogen bonding) between the analyte and the model molecules could inhibit the cross-linking aggregation. Some previous literatures have reported that heavy metal ion, melamine, or pesticides residues can be detected in food by using the principle of the blocking of gold nanoparticle aggregation. For example, the strong hydrogen bonding interaction between acetamiprid and chlorsulfuron will inhibit the AuNP aggregation induced by acetamiprid [119]. According to the above-mechanism, our group has proposed this anti-aggregation gold nanoparticle colorimetry to monitor low levels of chlorsulfuron in environmental water samples. The aggregation of gold nanoparticles could also be induced by melamine molecules due to its electron–rich nitrogen atoms in amino groups. Metsulfuron methyl will specifically bind to melamine molecules by forming hydrogen bonds. The inhibition of aggregation of gold nanoparticles could be applied for quantitative analysis of metsulfuron methyl residues in irrigation water samples [120], and the sensing principle for metsulfuron-methyl was shown in Figure 6. Anti-aggregation sensors are powerful techniques to detect mercury pollution. For instance, 2-mercaptobenzothiazole has the ability to induce gold nanoparticles aggregation by forming cross-linkages through Au–S bonds. While the presence of mercury(II) ions, the chelation interaction between 2-mercaptobenzothiazole and mercury(II) ions will strongly hinder its ability to induce the aggregation of gold nanoparticles. By utilization of the principle, mercury ions could be detected in environmental water samples and powdered milk [121].

Zhou et al. [122] found that Pb^2+^ could induce the aggregation of gold nanoparticles and cause the color of solution changed from red to blue. By taking advantage of the chelating reaction between Pb^2+^ and glyphosate, glyphosate can block the above aggregated process and the color of solutions remains wine-red. The limit of detection with the naked eye was 0.5 μM, and that with UV-visible spectroscopy was 2.38 nM. Melamine can induce gold nanoparticle aggregation and color changes. However, methionine can specifically bind with melamine through hydrogen bonds, and inhibit aggregation. An anti-aggregation colorimetric sensor has been successfully applied in a rapid test for methionine in plasma and urine with a linear range of 0–1.0 μM and a limit of detection of 24.5 nM [123].

To realize quantitative determination, gold nanoparticle colorimetric sensors can be produced by controlled reduction of gold nanoparticles by the target compound. He et al. [124] found that the benzene ring of a β-agonist, which possesses an amino or phenolic hydroxyl group, can be used directly to reduce chloroauric acid to gold atoms. The gold atoms then form gold nanoparticles, resulting in a characteristic peak at 528 nm with an absorbance proportional to the content of β-agonist. Methanobactin can reduce Au(III) to Au(0), which then forms gold nanoparticles, leading to a color change from yellow to red. When melamine is added, the oxazolone ring in methanobactin interacts with melamine to block the formation of gold nanoparticles. Melamine can also cause nanoparticle aggregation and a color change, which has been used in a colorimetric sensor for melamine in milk with a limit of detection of 0.238 μM [125]. Rawat et al. [126] used niacin to reduce Au(III) and prepare gold nanoparticles (ø 13.1 nm). The maximum emission wavelength was 534 nm. In the presence of arginine, histidine, methionine, or tryptophan, electrostatic interactions occurred and result in aggregation and a color change of the gold nanoparticles from wine red to blue. The linear range was 1–100 μM and the limits of detection were 7.2 nM for arginine, 4.2 nM for histidine, 5.1 nM for methionin, and 6.5 nM for tryptophan. In Table 1, the analystes, LOD and the detection strategies of in some reported AuNPs sensors were summarized.

## 6. Conclusions and Prospects

Compared with traditional methods, gold nanoparticle-based colorimetry possesses high sensitivity, and it does not need expensive equipment, and are simple. Relevant studies are continuously conducted in this area. However, to date, these studies have mainly focused on theoretical research and laboratory studies. There are issues with gold nanoparticle colorimetry that need to be resolved. First, gold nanoparticle-colorimetry is vulnerable to matrix interference from food samples such as vegetables, fruits and oil. Therefore, it is needed to develop the combined use of sample preparation techniques with gold nanoparticle-colorimetry by extracting analytes from complex food samples and reducing matrix interference. Second, the gold nanoparticles should be modified to bind specifically with the target compound, which will cause gold nanoparticle cross-linking and avoid aggregation caused by other factors. The design of ligands for nanoparticle modification should be considered. One direction is to screen for highly-specific aptamers that have high affinities with target contaminants, and another is to design specific ligands that bind through hydrogen bonds or electrostatic interactions. Furthermore, the sensitivity of colorimetry is relatively low compared with other methods, such as fluorescence, and signal amplification should be considered to improve the sensitivity for detection of substances present at low concentrations.

Overall, gold nanoparticle colorimetry methods are simple and rapid and do not require complicated equipment. Therefore, they can be applied widely for rapid testing, especially in food safety screening. Gold nanoparticle colorimetric technology is an important component of food quality and safety protocols. It is of great importance to develop new optical sensors based on functional gold nanoparticles for further development of the rapid test technology its applications in food quality and safety analysis.

## Figures and Tables

**Table 1:** Applications of colorimetric strategy based on AuNPs for food contaminants detection.

Type of Food Contaminants	Analytes	Colorimetric Strategy based on AuNPs	LOD	References
Heavy metals	Pd^2+^	Cross-linking	4230 nM	[75]
	Pb^2+^	Cross-linking	1.54 nM	[105]
	Cd^2+^	Cross-linking	3.5 nM	[106]
	Hg^2+^	Cross-linking	2.8 nM	[106]
	Cr^3+^	Cross-linking	10 nM	[107]
	As^3+^	De-protection	10 nM	[112]
	Hg^2+^	Anti-aggregation	6.0 nM	[121]
	Pb^2+^	Anti-aggregation	2.38 nM	[122]
Pesticides residues	Atrazine	Cross-linking	165 nM	[99]
	Dithiocarbamate	Cross-linking	1.05 nM	[100]
	Triadimenol	Cross-linking	182 nM	[101]
	Imidacloprid	De-protection	500 nM	[109]
	Cyromazine	De-protection	12 nM	[110]
	Chlorsulfuron	Anti-aggregation	70 nM	[119]
	Metsulfuron methyl	Anti-aggregation	131 nM	[120]
Veterinary drugs	Clenbuterol; Ractopamine	Cross-linking	0.23 nM; 0.43 nM	[103]
	Ractopamine; Salbutamol	Cross-linking	100 nM	[104]
	Sulfadimethoxine	De-protection	179 nM	[114]
	Ampicillin	De-protection	4.9 nM	[115]
	Oxytetracycline	De-protection	25 nM	[116]
	Melamine	Cross-linking	5.1 nM	[102]
	Melamine	Anti-aggregation	238 nM	[125]
